# Opinion of patients with ankylosing spondylitis on risk factors impairing their quality of life

**DOI:** 10.1007/s00296-012-2505-2

**Published:** 2012-09-15

**Authors:** Eugene J. Kucharz, Anna Kotulska, Magdalena Kopeć-Mędrek, Małgorzata Widuchowska

**Affiliations:** Department of Internal Medicine and Rheumatology, Medical University of Silesia, Katowice, Poland

**Keywords:** Ankylosing spondylitis, Patients’ opinion, Quality of life

## Abstract

We studied 54 patients with ankylosing spondylitis with questionnaire in order to determine their view on threat to quality of their life related to the disease. We have show that pain and significant disability are the main threats associated with the disease in view of the patients. Social aspects (losing of job or decreasing of income) are also important for the patients, while management of the disease is not considered as arduous. The results of patients’ opinion may be helpful in designing of educational programs for them.

## Introduction

Ankylosing spondylitis (AS) is a chronic, incurable, inflammatory disease affecting mainly the axial spine and sacroiliac joints [1]. The disease has a significant impact on patients’ quality of life [2–4]. A wide range of symptoms like pain, stiffness of joints and functional impairment profoundly affect physical, psychological and social state of the patients.

The aim of the present study was to obtain patients’ opinion on factors associated with AS that may potentially influence their quality of life. In other words, we evaluated threats to patients’ life associated with AS, which were considered by the patients as important risk factors.

## Materials and methods

The study was based on 54 patients (42 men, 12 women) with definite AS. The patients were associated with “the Pro Rheumate Group of AS patients,” a patient association in Katowice. In order to investigate the patients’ opinion, we created a questionnaire consisting of 12 questions. All questions had a form: “How important is to you a possible impact on your life of ….?” The patients were able to choose one of five answers, from “very important” to “not important at all.” The questions were presented in a mixed order but were focused on ability to work (and obtain income), family and social life as well as outcome of the disease (need for help by another person for everyday live activity, etc.). We also asked about importance of pain, frequent admissions to a hospital or impediments associated with management of AS. The questionnaire was anonymous and voluntary and was distributed at the patients’ group meeting. We limited demographic questions to sex of the patient, age (lower than 30 years, 31–45 years, over 45 years) and duration of AS symptoms (less 5 years, 6–10 years, over 10 years) in order to keep anonymity.

Fifty-four patients returned the questionnaire, and we scored the answers as following: very important = 4, important = 3, moderate important = 2, rather not important = 1, not important at all = 0.

## Results and discussion

**Fig. 1:**
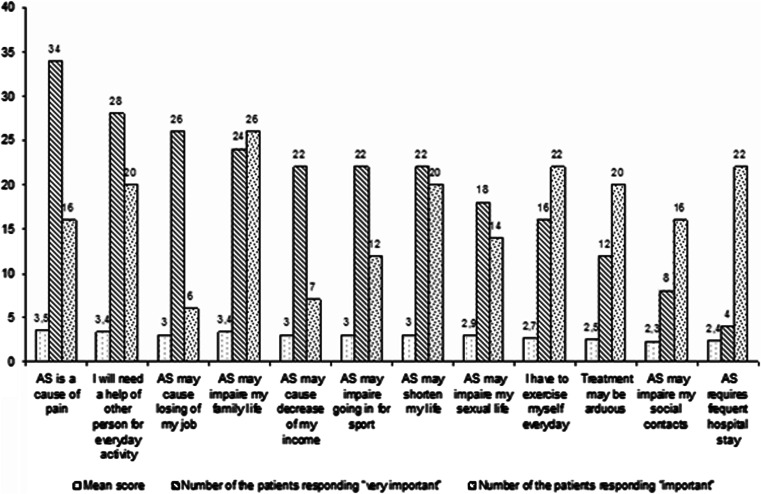
As patients’ option on importance of various factors potentially affecting their quality of life

Results are presented in Fig. 1. Pain is the main thread to the patients, and almost two-thirds of them consider pain as a very important risk factor to their quality of life. Problems in family life are also highly rated. More than a half of the patients were found to feel endangered by risk of needing help of another person for everyday functioning. It suggests that family life and help to a sick family member are closely associated, especially that there is a small tradition to use an institutional assistance for chronic patients in Poland. In most cases family feels responsible for help to a chronically disabled member. Social situation is also considered as endangered by AS. The mean score for losing a job or decreasing of income due to AS is 3.0 (important) and for almost a half of the investigated patients is very important. It may be associated with the fact that AS affects mostly young men that are in age of forming their own families.

Management of AS seems to be rather comfortable to the patients. Potential frequent hospitalizations were not considered as a significant factor impairing patients’ life. Some patients seem to have problems with performing home-based exercises everyday [5]. Going in for a sport is a problem for the patients, but it seems that the feeling of unfeasibility to go in for sport is more important for the patients than a real inability to participate in sport events. Thus, this is a kind of psychological distress caused by permanent disablement. Similar level of importance was shown for potential impairment in patients’ sexual life.

The main difference between our study and previous investigations is that we did not focus on quality of life of AS patients but on there views how disease may affect their life. Thus, our results reflect level of knowledge and understanding of the disease by the patients. It is important especially in chronic diseases. The questionnaire used for this study was created also for practical reasons. We would like to know more how the AS patients consider their “life with AS.” This knowledge is important for designing of the patient educational program.

Our study was based on 54 patients only. Due to low size of the investigated group, we did not analyze influence on patients’ opinion of such factors as age or duration of AS symptoms. It should be mentioned that some differences were found. Young male patients were more threaded by risk of losing of their jobs, while an impairment of sexual life due to the disease was more important to male patients than to female ones, but there was no relationship of the significance of the impairment with age of the patient. We do not collect data for analysis of relationship of education, place of living (city vs. country) and other factors to the patients’ view. Almost all our patients were living in a city, and all were treated with biologics. The other interesting aspect of the study is relationship of the patients’ view to their psychological state, and the threads may reflect not only patients’ knowledge of the disease but also their depression [6, 7].

Further studies in this field are needed to facilitate designing of educational programs for the AS patients.
